# Systems-level integration of machine learning, SHAP explainability, PCA, and network topology reveals multitarget protective actions of tannic acid against doxorubicin toxicity in pentose phosphate pathway

**DOI:** 10.1007/s00210-026-05236-6

**Published:** 2026-03-25

**Authors:** Betül Altunbilek, Duygu Kizir, Melike Karaman, Hamid Ceylan, Yeliz Demir

**Affiliations:** 1 Department of Molecular Biology and Genetics, Faculty of Science, Atatürk University, 25240 Erzurum, Turkey; 2 East Anatolian High Technology Research and Application Center (DAYTAM), Atatürk University, 25240 Erzurum, Turkey; 3 Department of Pharmacy Services, Nihat Delibalta Göle Vocational High School, Ardahan University, 75700 Ardahan, Turkey; 4 Department of Chemistry, Faculty of Science, Atatürk University, 25240 Erzurum, Turkey

**Keywords:** Doxorubicin, Gene expression, Pentose phosphate pathway, Tannic acid, Machine learning

## Abstract

Doxorubicin (DOX) is an effective chemotherapeutic agent; however, its clinical use is limited due to dose-dependent toxicities. This study aimed to investigate the potential protective effect of tannic acid (TA), a natural polyphenol with antioxidant properties, against DOX-induced alterations in oxidative pentose phosphate pathway (PPP) enzymes in rat lung tissue. Male rats were treated with DOX, TA, or a combination of both. The activity and gene expression levels of PPP enzymes (G6PD and 6PGD), antioxidant enzymes (GPx, SOD, and CAT), and oxidative stress markers (GSH and MDA) were evaluated. DOX administration significantly reduced G6PD and 6PGD activity and gene expression, decreased GSH levels, and increased MDA content. Co-treatment with TA reversed these biochemical alterations and improved antioxidant status. To elucidate the mechanistic drivers of group discrimination, an explainable random forest classifier was developed. SHAP (Shapley Additive Explanations) analysis identified 6PGD activity, G6PD/6PGD mRNA expression, GPX and CAT activities, IL-6 mRNA, GSH, and MDA as the most influential biomarkers determining DOX toxicity profiles. In addition to conventional biochemical and molecular assessments, multivariate statistical analyses (PCA), correlation network mapping, and explainable machine learning approaches (random forest with SHAP analysis) were employed to characterize the systemic oxidative and inflammatory alterations induced by DOX and their modulation by TA. The classifier achieved excellent discriminative performance, with a low out-of-bag Brier score (0.138). TA demonstrates a protective effect against DOX-induced oxidative stress and enzymatic impairment in the rat lung, suggesting its potential as a supportive therapeutic agent.

##  Introduction

Doxorubicin (DOX) is a widely used anthracycline antibiotic with broad-spectrum antitumor activity (Li et al. 2022; Kizir et al. 2024). It is commonly administered in the treatment of various cancers, including hematologic malignancies and solid tumors (Kaymak et al. 2021; Özturk et al. 2024). However, its clinical application is significantly limited by cumulative dose-dependent toxicities, particularly cardiotoxicity (Zhao and Zhang 2017), nephrotoxicity (Lahoti et al. 2012), and pulmonary toxicity (Prasanna et al. 2020). These adverse effects are predominantly mediated by oxidative stress through the excessive generation of reactive oxygen species (ROS), leading to cellular injury, lipid peroxidation, DNA damage, and apoptosis (Beydemir and Demir 2017; Türkan et al. 2019; Demir and Köksal 2019).

Among various organ systems affected by DOX-induced oxidative injury, the lung has received relatively less attention in preclinical investigations, despite being a critical site of oxygen exchange and oxidative metabolism. The lung’s high oxygen environment makes it particularly vulnerable to ROS-mediated damage, especially when antioxidant defenses are compromised (Owumi et al. 2021; Erdem Guzel and Kaya Tektemur 2021). The pentose phosphate pathway (PPP), with its key enzymes glucose-6-phosphate dehydrogenase (G6PD) and 6-phosphogluconate dehydrogenase (6PGD), plays an essential role in maintaining redox balance by producing NADPH, which is required for glutathione recycling. Inhibition of these enzymes impairs antioxidant capacity and promotes oxidative damage (Yıldız et al. 2022; Demir et al. 2024).

Tannic acid (TA), a polyphenolic compound found in many plants and foods, has been widely studied for its antioxidant, anti-inflammatory, antimicrobial, and anticancer properties (Baldwin and Booth 2022). Its ability to scavenge free radicals and modulate redox-sensitive signaling pathways makes TA a promising candidate for mitigating drug-induced oxidative damage. Recent studies have demonstrated the protective effects of TA in models of DOX-induced toxicity, including nephrotoxicity and hepatotoxicity (Kaymak et al. 2021; Yesilkent and Ceylan 2022). However, to the best of our knowledge, no study has specifically investigated the effect of TA on the activity and gene expression of PPP enzymes in the context of DOX-induced pulmonary toxicity.

Therefore, the study was designed to assess whether TA mitigates DOX-induced pulmonary oxidative and inflammatory responses. The DOX group served as a positive control to establish the injury/inflammation phenotype against which TA’s protective effects were evaluated. In addition, we assessed the impact of TA on antioxidant enzyme levels (SOD, CAT, and GPx), oxidative stress markers (MDA, GSH), and proinflammatory cytokines (TNF-α, IL-6). The findings of this study are expected to contribute to the growing body of evidence supporting the use of natural antioxidants as adjuncts in cancer therapy to reduce chemotherapy-associated organ toxicities.

## Materials and methods

### Procedure for experiments and animal care

The rats (male *Rattus norvegicus*, 180 ± 20 g, *n* = 20) utilized for the current research were supplied by the Atatürk University Medical Experimental Application and Research Unit. The Local Ethics Committee for Animal Experiments at Atatürk University permitted the employing of animals based on the National Institutes of Health’s Animal Research directives (Protocol No. 2021/3–65). Only male rats were used in this study to reduce hormonal variability related to the estrous cycle that may confound oxidative and inflammatory outcomes and to maintain a controlled proof-of-concept design. Rats were maintained in typical ambient conditions (12-h light 12-h dark light cycle, 40–60% humidity, 22 ± 1 °C) and offered unlimited access to food and water. Four rat groups were chosen at random:

- Control group (Con): Rats received intraperitoneal (i.p.) injections of normal saline for 14 consecutive days.
- Tannic acid group (TA): Rats were administered tannic acid (TA, 50 mg/kg, i.p.) for 14 consecutive days (Yesilkent and Ceylan 2022; Kizir et al. 2023).
- Doxorubicin group (DOX): Rats received DOX via six equal i.p. injections of 5 mg/kg each to achieve a cumulative dose of 30 mg/kg over the experimental period. To equalize the stress associated with injections, normal saline (i.p.) was administered on the non-DOX injection days (Demir et al. 2026).
- Combined treatment group (DOX + TA): Rats received both TA and DOX according to the regimens described above (Groups 2 and 3). Specifically, TA (50 mg/kg, i.p.) was administered 1 h before each DOX injection, and saline (i.p.) was administered on non-DOX injection days to match handling/injection frequency.

After treatment, rats were sacrificed using a ketamine/xylazine (3:1) cocktail (Yesilkent and Ceylan 2022). For analysis, lung tissues were immediately retrieved and held at − 80 °C.

### Molecular analysis protocols

According to the manufacturer’s instructions, 30 mg of the rat lung tissue was employed to gather total RNA through EcoPURE RNA kit (EcoTech). Both the purity and amount of the RNA were evaluated utilizing a NanoDrop spectroscopy (Thermo Scientific, Multiskan GO, USA). The iScript™ cDNA Synthesis Kit (Bio-Rad) was implemented to generate cDNA from 1 µg of total RNA in line with the directions given by the manufacturer. Under the manufacturer’s instructions, the Rotor-Gene Q equipment (Qiagen) was employed to carry out the quantitative real-time PCR utilizing SsoAdvanced Universal SYBR Green Supermix (Bio-Rad). The set of primer sequences employed in gene expression is listed in Table 1. The 2^(−∆∆CT)^ procedure was applied to estimate the relative shifts in gene expression (Livak and Schmittgen 2001). With the goal to normalize data on gene expression, Gapdh functioned as a housekeeping gene.

**Table 1:** The set of primer sequences utilized for qRT-PCR

Genes	5′−3′ forward primers	5′−3′ reverse primers	References
*G6pd*	GCGTATCTTCACACCACTGC	AGCCCACTCTCTTCATCAGC	(Sun et al. 2019)
*6pgd*	GGCCATCGCTGCAAAAGTAG	TCTTCAAACGCCTGAGCCAT	(Wang et al. 2018)
*Sod*	GGTCCACGAGAAACAAGATGA	CAATCACACCACAAGCCAAG	(Dong et al. 2018)
*Cat*	ACATGGTCTGGGACTTCTGG	CCATTCGCATTAACCAGCTT	(Zhu et al. 2019)
*Gpx*	TCGGACATCAGGAGAATGG	AGGTAAAGAGCGGGTGAGC	(Yesilkent and Ceylan 2022)
*Tnf-α*	AGGAGGGAGAACAGCAACTC	TGTATGAGAGGGACGGAACC	(Lu et al. 2021)
*Il-6*	AGTTGCCTTCTTGGGACTGA	TACTGGTCTGTTGTGGGTGG	(Yesilkent and Ceylan 2022)
*Gapdh*	AAACCCATCACCATCTTCCA	ATACTCAGCACCAGCATCACC	(Yesilkent and Ceylan 2022)

### Preparation of tissue homogenates and determination of protein content

The lung tissues were homogenized utilizing Tris–HCl buffer (50 mM containing 1 mM DDT, 1 mM EDTA, and 1 mM PMSF—pH 7.6) in TissueLyser LT instrument (Qiagen). After centrifuging the homogenates, the supernatants were collected for the experiments. Bradford’s method was implemented to spectrophotometrically identify protein contents of the tissues (Bradford 1976).

### Determination of enzyme activities

The activities of G6PD and 6PGD enzymes were determined spectrophotometrically as described by Beutler (Beutler 1971). The quantity of enzyme needed for reducing 1 µmol of NADP + in 1 min was stated by one unit of enzyme. The colorimetric procedure suggested by Sun et al. (Sun et al. 1988) was implemented to assess the activity of superoxide dismutase (SOD). The quantity of enzyme that suppresses 50% of the rate of nitroblue tetrazolium reduction was expressed as one unit of SOD. Aebi (Aebi 1984) approach was followed for determining the activity of catalase (CAT). The quantity of enzyme needed for converting 1 µmol H_2_O_2_ per minute was defined as one unit of CAT. The method cited by Beutler (Beutler 1984) was applied for measuring glutathione peroxidase (GPx). The quantity of enzyme needed to oxidize one µmol of NADPH per minute was identified to one unit of GPx.

### Determination of malondialdehyde and reduced glutathione levels

One of the most recognized indicators in lipid peroxidation procedures is malondialdehyde (MDA). The spectrophotometric approach described by Ohkawa (Ohkawa et al. 1979) was utilized for determining the MDA level. Absorbance measurements recorded at 532 nm were evaluated from a standard curve established with 1,1,3,3-tetraethoxypropane (Sigma-Aldrich). The content of reduced glutathione (GSH) was quantified as explained by Ellman (Ellman 1959). The concentrations of GSH were calculated from a standard curve adjusted using L-glutathione reduced (Sigma-Aldrich).

### Machine learning and SHAP analysis

Machine learning analysis was performed as a complementary multivariate approach to integrate all endpoints simultaneously and to identify the most informative variables distinguishing the groups. Model performance was assessed under cross-validation, and feature contributions were interpreted using SHAP-based attribution to prioritize key biomarkers driving the DOX-injury signature and its attenuation by TA.

A probabilistic random forest classification model containing 500 trees and using the Gini split rule was constructed to determine the relative importance of biochemical and molecular markers to toxicity due to DOX. Out-of-bag (OOB) error estimates were used to internally evaluate model performance, whereas a 70/30 train-test split was used to evaluate it externally. Attributes of the features were performed using Shapley Additive Explanations (SHAP) that quantify the marginal contribution of a single biomarker to individual predictions; aggregate importance was described as mean absolute SHAP values. Stability and robustness were also explored through permutation importance, where every feature is irregularly rearranged to ascertain its impact on prediction error. All analyses were performed in R (version 4.5.1, R Foundation for Statistical Computing, Vienna, Austria) using the ranger, fastshap, and ggplot2 packages.

### Model validation

In order to ensure the stability of the machine learning model and the interpretability generated by the model, different validation approaches were used. One, internal performance was measured with the help of OOB estimates made by the random forest classifier. Second, external 70/30 train-test partition was done to test the generalizability of the model. Third, the importance of permutation analysis was used to confirm the robustness of influence by features by measuring the reduction in predicting accuracy after each biomarker was randomly shuffled. Lastly, model discrimination has been studied with the aid of confusion matrix analysis and one-vs-rest receiver operating characteristic (ROC) curves. The agreement between SHAP attribution values and rankings of permutation importance was used as another criterion to support the robustness of interpretability.

### Principal component analysis (PCA)

PCA was performed to query the overall architecture of the experimental biomarkers of biochemical and gene expression between the experimental cohorts (control, DOX, TA, and DOX + TA). All the variables were scaled and centered before analysis. The R (version 4.5.1, R Foundation for Statistical Computing, Vienna, Austria) was used to create PCA score plots, and 600-dpi vector images were exported to guarantee publication-level quality visualization. The tendency of group separation and clustering was tested by using PC1 and PC2 that accounted for most of the variance in the dataset.

### Correlation heatmap

Pairwise relationships among biochemical parameters and gene expression markers were assessed using Pearson’s correlation coefficients. Prior to analysis, all variables were inspected for normality and standardized (z-score transformation) to ensure comparability across different measurement scales. A correlation matrix was computed, and hierarchical clustering was applied to both rows and columns to identify co-regulated biochemical modules. The resulting correlation heatmap was generated using the pheatmap package in R (version 4.5.1, R Foundation for Statistical Computing, Vienna, Austria), employing Euclidean distance and complete-linkage clustering. Strong positive and negative correlations were highlighted through a color-scaled gradient, enabling visualization of coordinated changes induced by DOX and their modulation by TA.

### Correlation network analysis

A correlation network was created using ipath (R version 4.5.1, R Foundation for Statistical Computing, Vienna, Austria) in order to visualize relationships between markers. Connection lines were made where the variables had a correlation of 0.6, and the width of the lines was adjusted to display the size of the correlation. The green and red colors represented positive and negative correlations, respectively. The topology of the network helped to identify functional biochemical modules that were modified by DOX and reinstated by TA.

### Statistical analysis

A statistical analysis software (GraphPad Software 8.0, LLC, San Diego, CA, USA) was implemented for assessing the data. Means ± SEM were employed to express all data. Tukey’s multiple comparison tests were utilized after an analysis of variance (ANOVA) to find out the differences between the groups. A statistically significant difference was interpreted as a *p* value of less than 0.05. Asterisks *, **, and *** indicated the degree of significance at *p* < 0.05, 0.01, and 0.001, respectively.

## Results

### Effect of treatments on PPP enzyme activity and gene expressions (G6PD and 6PGD)

**Fig. 1:**
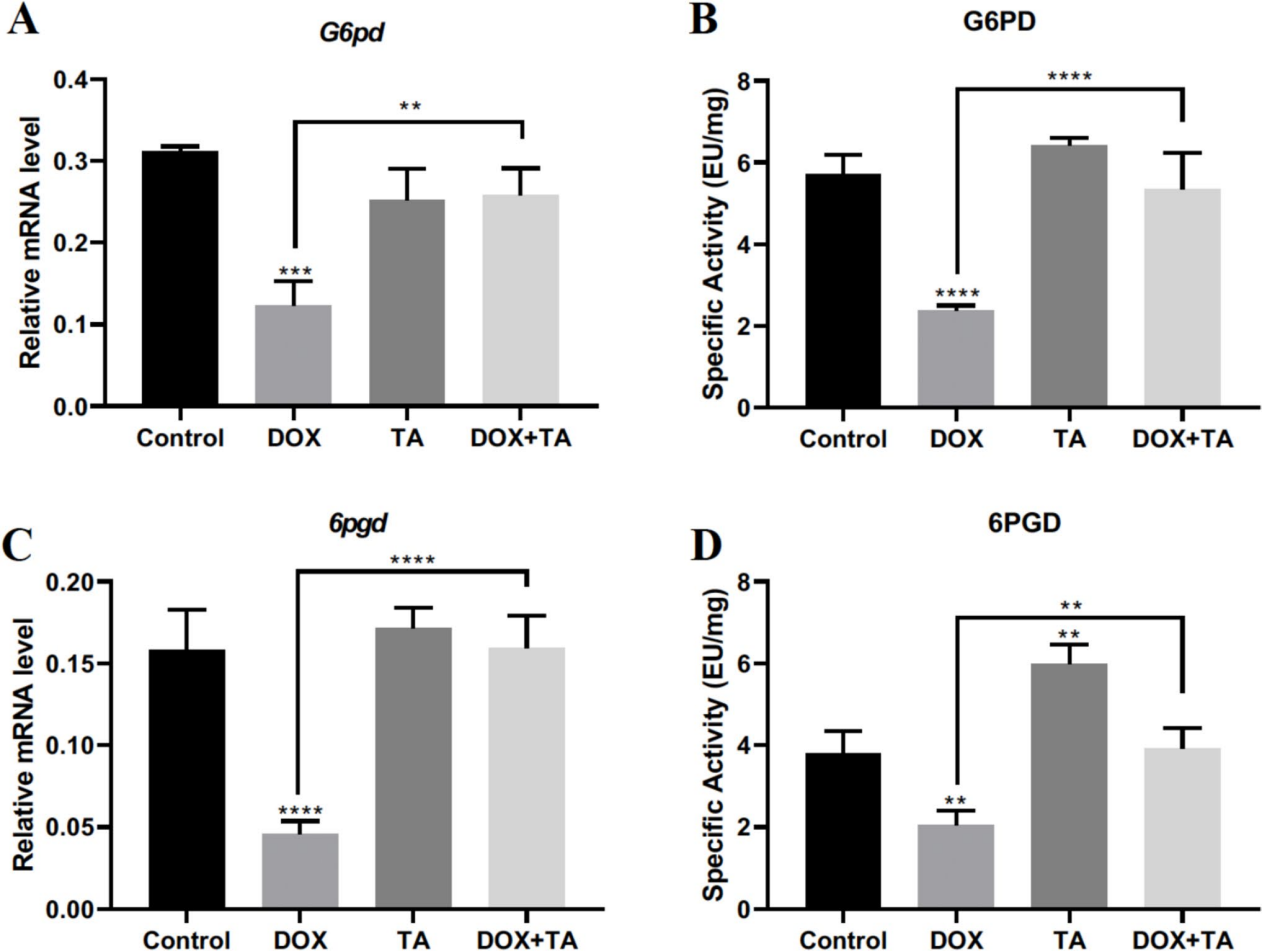
Influence of TA with respect to the relative expression level and activity for G6PD and 6PGD in rat lung that received DOX and/or TA. Data are presented as mean ± SEM (*n* = 5 rats per group; 20 male *Rattus norvegicus*, aged 8–10 weeks, 180 ± 20 g). Statistical analysis was performed using one-way ANOVA followed by Tukey’s post hoc test, which accounts for multiple comparisons. ^*^p < 0.05 was considered statistically significant. TA: tannic acid, DOX: doxorubicin, G6PD: glucose-6-phosphate dehydrogenase, 6PGD: 6-phosphogluconate dehydrogenase

Compared to the control group, DOX treatment significantly reduced both the mRNA expression and enzymatic activities of G6PD and 6PGD in rat lung tissues (*p* < 0.05). Co-administration of TA with DOX significantly reversed these reductions (*p* < 0.05), restoring enzyme levels close to control values. Notably, TA treatment alone also caused a moderate increase in 6PGD activity (Fig. 1).

### Antioxidant enzyme activities (SOD, CAT, and GPx) and gene expressions

**Fig. 2:**
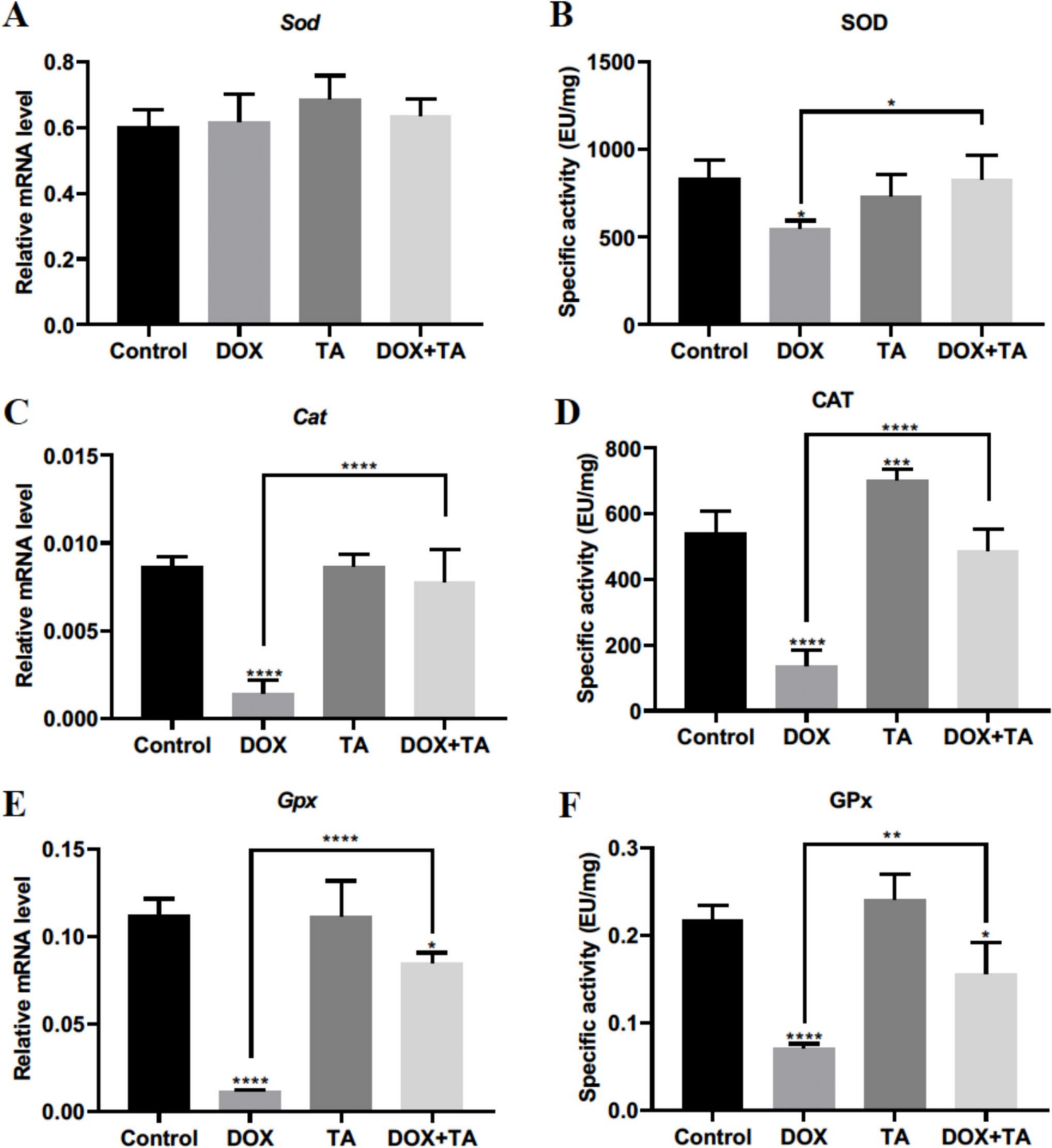
Influence of TA on the relative mRNA level and enzyme activity for SOD, CAT, and GPx in rat lung that received DOX and/or TA. Data are expressed as mean ± SEM (*n* = 5 rats per group; 20 male *Rattus norvegicus*, aged 8–10 weeks, 180 ± 20 g). One-way ANOVA with Tukey’s multiple comparison test was used for statistical analysis. Significance was set at ^*^p < 0.05. TA: tannic acid, DOX: doxorubicin, SOD: superoxide dismutase, CAT: catalase, GPx: glutathione peroxidase

Relative expression level and activity for the antioxidant enzymes in the lung tissue of rats given DOX and/or TA are depicted in Fig. 2. The SOD enzyme activity of the DOX group dropped statistically considerably (*p* < 0.05) in comparison to the control group, whereas the TA and TA + DOX groups did not differ statistically significantly (*p* > 0.05). In contrast with the control group, exposure to DOX treatment dramatically lowered the relative mRNA level and enzyme activity for CAT in lung tissue (*p* < 0.001). Interestingly, the DOX-mediated drop in CAT relative gene expression and activity in rat lung tissue was restored by TA therapy (*p* < 0.001). In comparison to the control, DOX treatment brought about a statistically significant decline in GPx relative gene expression and activity in lung tissue (*p* < 0.001). However, when DOX and TA were administered together, the DOX-mediated decline in rat lung tissue reached the control group’s relative gene expression and activity level.

### Malondialdehyde (MDA) and glutathione (GSH) levels

**Fig. 3:**
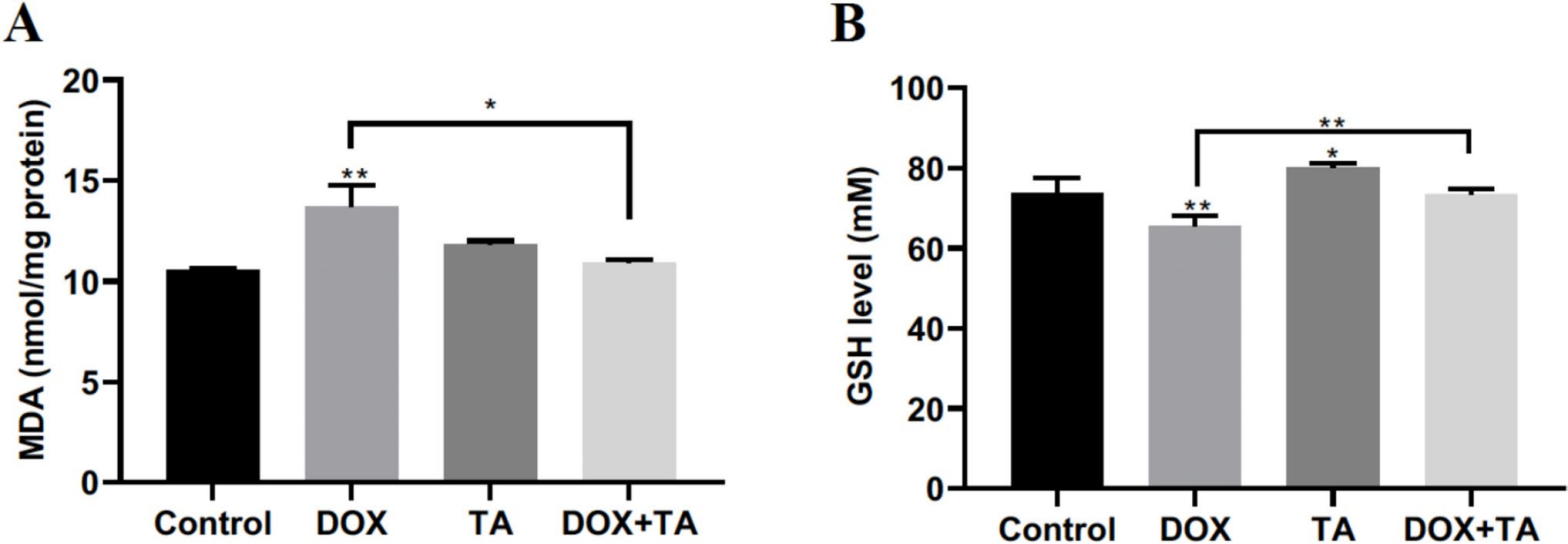
Influence of TA with respect to MDA and GSH levels in rat lung that received DOX and/or TA. Data represent mean ± SEM (*n* = 5 rats per group; 20 male *Rattus norvegicus*, aged 8–10 weeks, 180 ± 20 g). Statistical comparisons were performed by one-way ANOVA followed by Tukey’s post hoc test. ^*^p < 0.05 indicates statistical significance. TA: tannic acid, DOX: doxorubicin, MDA: malondialdehyde, GSH: reduced glutathione

Oxidative stress occurs when the oxidant-antioxidant balance in the cell shifts in favor of oxidants. MDA formed as a result of lipid peroxidation was analyzed as an oxidant indicator and GSH as an antioxidant indicator. The levels of MDA and GSH were determined in rat lung tissue. As shown in Fig. 3, MDA content elevated considerably after exposure to DOX in comparison with the control (*p* < 0.01), whereas it produced a significant drop in the amount of GSH (*p* < 0.01). DOX-mediated alterations in MDA and GSH levels in rat lung tissue were reversed when TA and DOX were received together.

### Inflammatory cytokine expression (TNF-α and IL-6)

**Fig. 4:**
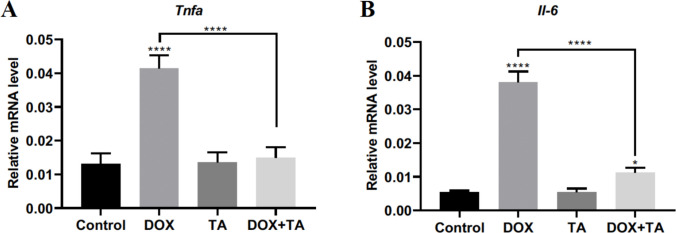
Influence of TA on Tnf-α and Il-6 relative gene expression levels in rat lung that received DOX and/or TA. Values are expressed as mean ± SEM (*n* = 5 rats per group; 20 male *Rattus norvegicus*, aged 8–10 weeks, 180 ± 20 g). Statistical significance was determined using one-way ANOVA followed by Tukey’s multiple comparison test. ^*^p < 0.05 was considered significant. TA: tannic acid, DOX: doxorubicin, Tnfa: tumor necrosis factor alpha, Il-6: interleukin 6

Furthermore, in treated rat lung tissues, the relative mRNA levels for interleukin-6 (Il-6) and tumor necrosis factor (Tnf-α), which are proinflammatory genes, were evaluated (Fig. 4). As expected for the established DOX injury model, Tnf-α and Il-6 were significantly upregulated in the DOX group; importantly, TA co-treatment significantly attenuated these increases (DOX + TA vs DOX).

### Machine learning analysis

**Fig. 5:**
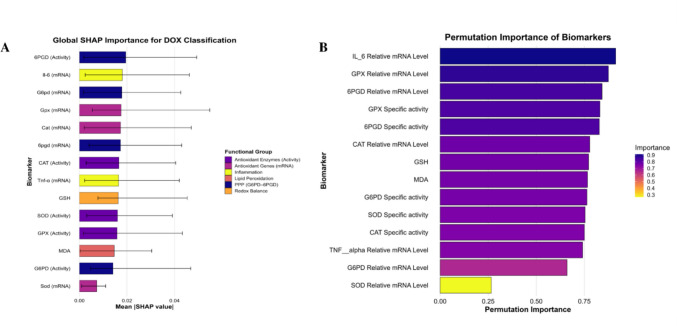
Machine learning interpretability analysis integrating SHAP and permutation importance. **A** Global SHAP importance showing the mean absolute contribution of each biomarker to DOX classification, categorized by functional group (PPP, antioxidant enzymes, antioxidant gene markers, inflammation, lipid peroxidation, redox balance). **B** Permutation-based importance ranking demonstrating the performance degradation upon random shuffling of each biomarker

A separate set of biomarkers that made a huge contribution to the classification of DOX-exposed groups was identified by machine learning analysis. Figure 5A suggests that SHAP global importance showed that 6PGD activity, G6PD/6PGD (mRNA), GPX and CAT activities, IL-6 and TNF-alpha (mRNA), GSH, and MDA had the greatest contributions to drive the model predictions. All of these characteristics suggest that pentose phosphate pathway (PPP) flux disruption, antioxidant activity impairment, and inflammatory signal activation are the central biochemical phenotypes of DOX toxicity.

In line with the SHAP results, permutation importance analysis (Fig. 5B) generated a much more stable overlapping biomarker hierarchy. The most influential predictors were IL-6 (mRNA) and GPX (mRNA and activity), 6PGD (mRNA and activity), CAT (mRNA), GSH, MDA, and G6PD (mRNA and activity) activity. The high correspondence between SHAP-generated attributions and importance using permutation is a guarantee of the good soundness of the classifier and the strength of trust in the biological relevance of the discovered markers. The interpretations were also supported by model performance measures. The independent test set was correctly classified by the classifier at 100% and the out-of-bag (OOB) Brier score was low (0.138), which suggests that the classifier has a good discriminative ability and issued good probability estimates. Combining these findings, the consistency between SHAP and permutation output and the high predictive power of the machine-learned framework used in this work confirm the stability and explainability of the machine learning framework used in the work (Fig. 5).

### PCA results

**Fig. 6:**
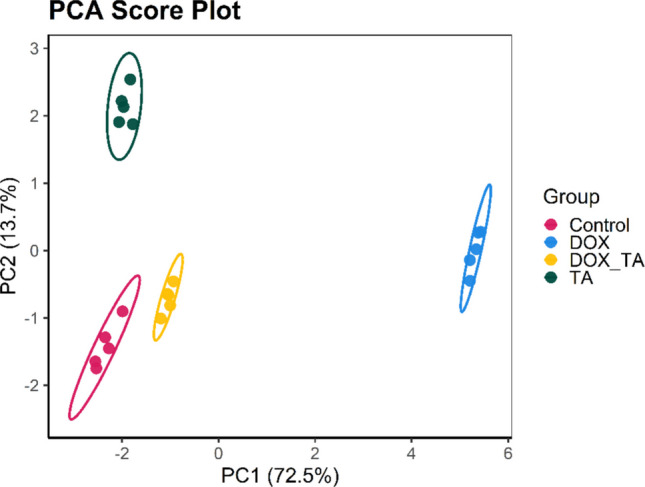
PCA score plot showing separation of groups along PC1 and PC2

PCA showed a clear difference between the DOX group and the other experimental groups along PC1 which showed that there was a distinct biochemical signature of the DOX-induced oxidative and inflammatory stress (Fig. 6). The TA and DOX + TA groups were closer to the control group, which indicated a partial recovery of the biochemical profile.

### Correlation heatmap

**Fig. 7:**
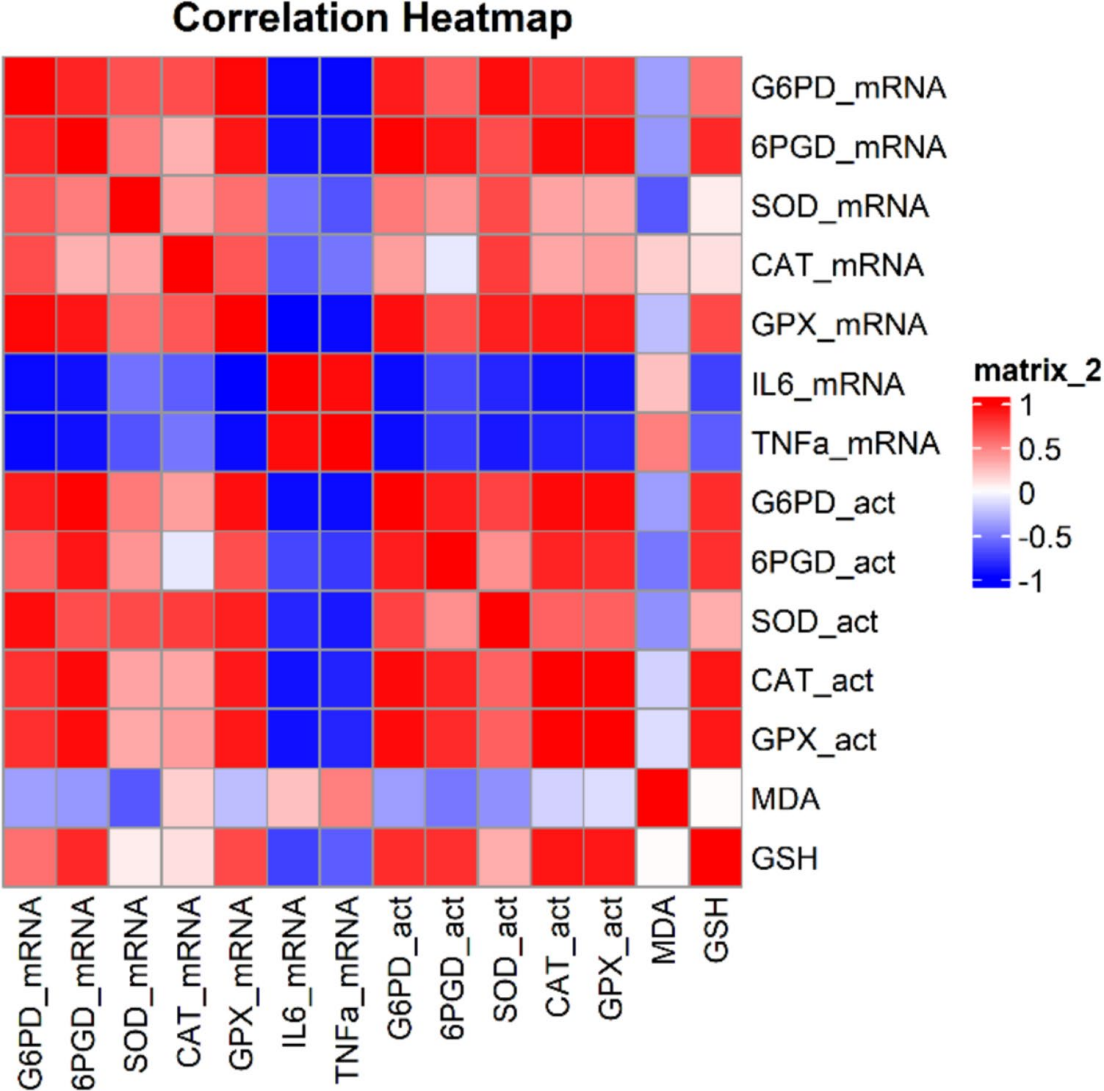
Correlation heatmap of biochemical and molecular markers

The correlation heatmap revealed two large clusters: (i) the activities of antioxidant enzymes and their mRNA equivalents and (ii) inflammatory cytokines (IL-6, TNF-a) and oxidative damage markers (MDA). DOX treatment broke the internal consistency of the antioxidant cluster and enhanced pathological relations among inflammatory biomarkers and MDA (Fig. 7).

### Correlation network

**Fig. 8:**
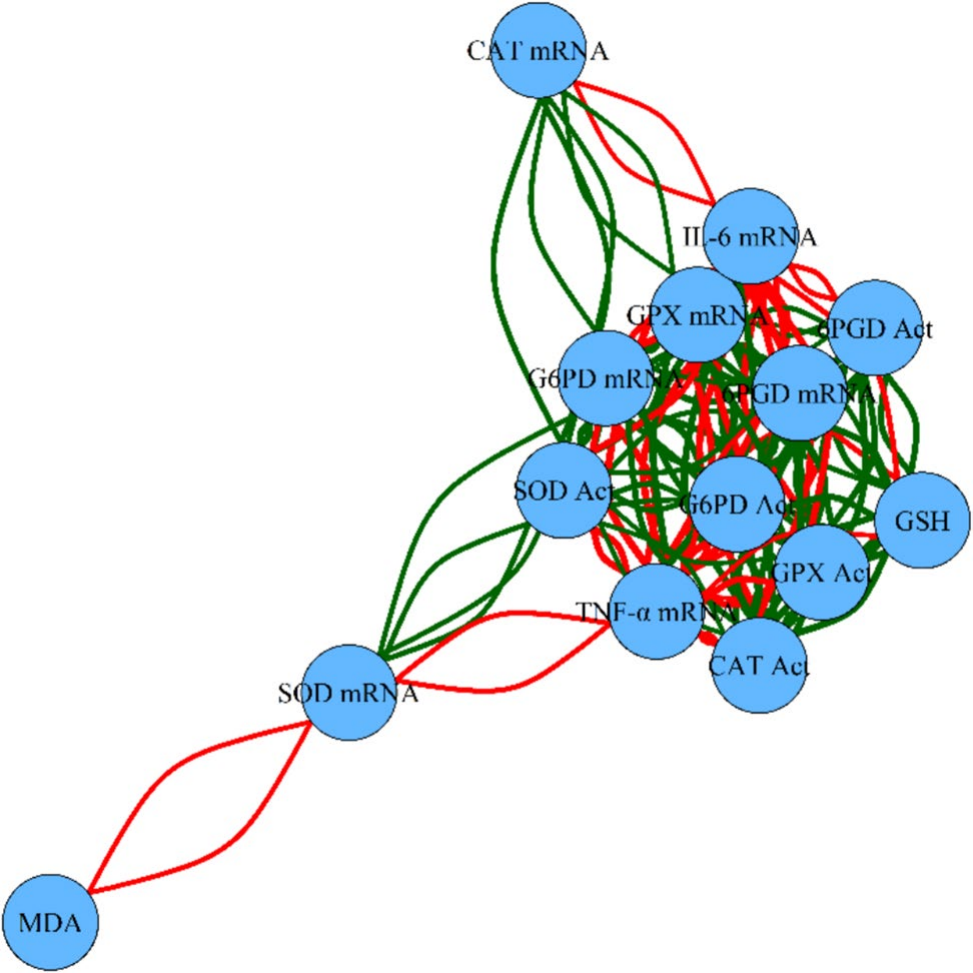
Correlation network illustrating positive (green) and negative (red) interactions among oxidative stress and inflammatory markers

The perturbations on the system caused by DOX were further illustrated in the correlation network. The nodes that showed weaker connectivity in the animals treated with DOX were antioxidant-related nodes (G6PD, 6PGD, SOD, CAT, and GPX), and inflammatory nodes were more strongly related. TA co-treatment redefined the antioxidant connectivity and reduced the intensity of the inflammatory relationship (Fig. 8), thus proving a universal biochemical recuperation method.

## Discussion

Multiple organ damage usually occurs during chemotherapeutic treatment of cancers (Sak 2012). The chemotherapeutic agent DOX, which is widely used in cancer treatment, possesses a number of undesirable consequences such as hepatotoxicity, cardiotoxicity, and nephrotoxicity. Several molecular mechanisms have been investigated in DOX toxicity, including inflammatory reaction, cellular oxidative stress, and apoptotic cell death. However, oxidative stress is the most emphasized mechanism (Mantawy et al. 2014). Therefore, studies investigating the potential protective effect of natural compounds with antioxidant properties to prevent DOX-induced oxidative stress have been focused (Mohajeri and Sahebkar 2018; Ekinci Akdemir et al. 2021). In this study, we demonstrated that DOX significantly impairs the activity and expression of oxidative PPP enzymes, as well as key antioxidant enzymes, in rat lung tissue. Moreover, DOX exposure resulted in elevated oxidative stress markers and proinflammatory cytokines. These findings confirm the oxidative and inflammatory nature of DOX-induced pulmonary toxicity. Notably, co-administration of TA reversed these alterations, highlighting its protective role against DOX-mediated lung damage.

The PPP is among of the basic antioxidant mechanisms for defense of cells (Riganti et al. 2012). Among the essential PPP enzymes are G6PD and 6PGD. They are the main supplier for the reduced nicotinamide adenine dinucleotide phosphate (NADPH), essential for many reduction reactions (Ulusu et al. 2003). Therefore, these enzymes are important in protecting cells against oxidative stress because NADPH produced by these enzymes detoxifies ROS and replenishes GSH via glutathione reductase (GR) action (Rosa et al. 2012).

This investigation revealed a diminished expression level and enzyme activity for G6PD and 6PGD in the lung tissues of DOX-exposed rats. Consequently, a decline in both mRNA expressions and activities for these enzymes sensitizes cells to oxidative stress. On the other hand, TA exposure in combination with DOX ameliorated DOX-elicited changes in these enzymes. TA’s reparative role may be attributed to its radical scavenging properties and antioxidant capacity. Furthermore, TA may support the maintenance of intracellular NADPH levels, an essential cofactor for GSH regeneration, by promoting PPP activity and potentially inhibiting NADPH-consuming enzymes such as NADPH oxidase. This effect sustains glutathione recycling and reinforces the intracellular redox buffer system (Rosa et al. 2012). Since PPP is a primary source of NADPH required for maintaining reduced GSH levels, inhibition of this pathway may contribute to cellular vulnerability under DOX-induced oxidative conditions. Our data suggest that TA mitigates this suppression, possibly by directly scavenging ROS or by restoring NADPH-dependent redox balance. Similar enzyme-preserving effects have been reported with other natural antioxidants such as vitamin E and gallic acid (Gumustekin et al. 2005; Ekinci Akdemir et al. 2021).

One of the main mechanisms underlying DOX-mediated cardiotoxicity is oxidative stress brought on by a high level of generated free radicals (Rawat et al. 2021). Free radical scavenging enzymes are involved in cellular defense against oxidative damage. GPx, SOD, and CAT, which are important antioxidant enzymes, fulfill a crucial role in mitigating or combating damage caused by reactive oxygen species (Ighodaro and Akinloye 2018). Therefore, since the balance between these enzymes is important in the removal of oxygen stress in intracellular organelles, the changes of these enzymes are examined in the prevention of DOX-induced toxicity. Lipid peroxidation is a hallmark of oxidative tissue injury. Oxidative stress can induce lipid peroxidation in biological samples, leading to the formation of harmful by-products such as MDA. Therefore, MDA is used as a biomarker to analyze oxidative stress-induced defects in a biological sample (Cordiano et al. 2023). Elevated MDA levels in DOX-treated rats indicate enhanced ROS-mediated damage to membrane lipids. The concomitant decline in GSH, a key intracellular antioxidant, further emphasizes the oxidative burden. TA’s capacity to normalize MDA and GSH levels suggests it effectively counters lipid peroxidation and supports intracellular redox buffering.

These outcomes align with findings from Kuzu et al. 2019 and Chen et al. 2016, who reported comparable biochemical improvements following antioxidant administration specifically (Morin in Kuzu et al.) and (Berberine in Chen et al.) in DOX-induced organ injury models. In cardiac tissue, DOX-mediated cardiotoxicity revealed elevated MDA levels and diminished GSH, SOD, and CAT activity. Naringin exposure improved changes in the antioxidant enzymes as well as GSH and MDA levels (Kwatra et al. 2016). The current investigation revealed similar findings. TA enhanced the DOX-mediated drop in MDA levels and diminished the DOX-mediated rise in GSH, GPx, SOD, and CAT values. By correcting the alterations in antioxidant system characteristics, all of these data suggest that natural substances with antioxidant qualities have the ability to protect against a variety of toxicities caused by DOX. Compared with naringin, TA is a hydrolysable tannin with a high density of phenolic hydroxyl groups, supporting strong radical-scavenging and metal-chelating capacity. Given that DOX toxicity is closely linked to oxidative mechanisms, these chemical features provide a plausible rationale for TA’s protective profile and justify its investigation as a complementary candidate. Future studies should include dose–response and head-to-head comparisons to position TA relative to other natural agents.

The protective effect of TA against DOX-induced oxidative and inflammatory damage has been clearly demonstrated in our study. However, the molecular mechanisms by which TA exerts these effects deserve further elaboration. In the literature, TA’s antioxidant and cytoprotective activities have been attributed not only to its direct radical scavenging capacity but also to its ability to modulate redox-sensitive cellular signaling pathways (Nagesh et al. 2020). Foremost among these is the activation of the Nrf2/Keap1 signaling pathway (Kanner 2020). Nrf2 (nuclear factor erythroid 2–related factor 2) is a master regulator of antioxidant defense. Under oxidative stress conditions, TA is believed to alter Keap1 conformation, thereby promoting the nuclear translocation of Nrf2 (Li et al. 2020; Liu and Guo 2024). This, in turn, induces the expression of antioxidant enzymes such as G6PD, 6PGD, SOD, CAT, and GPx, which are essential for cellular redox homeostasis (Riganti et al. 2012; Baldwin and Booth 2022). Additionally, TA has been shown to affect MAPK (mitogen-activated protein kinase) signaling pathways, particularly ERK1/2 and p38 MAPK, which play vital roles in adaptive responses to oxidative stress (Sivanantham et al. 2019). Through modulation of phosphorylation dynamics of these kinases, TA can promote cytoprotection and suppress oxidative damage (Zhu et al. 2019).

Inflammation plays a synergistic role in DOX toxicity by amplifying ROS generation and triggering cytokine cascades. The upregulation of TNF-α and IL-6 in our study is consistent with the known proinflammatory effects of DOX. According to one study, IL-6, IL-1β, and TNFα, the proinflammatory cytokines, were found to be considerably enhanced in response to DOX-induced cardiorenal injury (Xing et al. 2022). Furthermore, it was shown that costunolide, which occurs naturally in Compositae plants, decreased the rise in expression levels of the proinflammatory cytokines (Xing et al. 2022). On the other hand, naringenin exposure in combination with DOX has also been reported to elicit anti-inflammatory properties through lowering the expression levels of these inflammatory markers (Subburaman et al. 2014). Another critical mechanism of TA is the inhibition of the NF-κB pathway. DOX-induced oxidative stress is known to activate NF-κB, resulting in increased expression of proinflammatory cytokines such as TNF-α and IL-6 (Ibrahim Fouad and Ahmed 2022). Present research revealed that the DOX-mediated rise in *Tnf-α* and *Il-6* gene expression detected in rat lung tissues was mitigated by TA treatment. Similarly, TA diminished proinflammatory cytokine expression levels, which were noticeably enhanced in kidney injury triggered by DOX (Yesilkent and Ceylan 2022). Our findings support this mechanism, as TA co-treatment significantly reduced DOX-induced cytokine gene expression in lung tissues. All these findings suggest that TA interferes with immunity by disrupting the expression level of inflammation-related genes in DOX-induced toxicity. Furthermore, the use of bioactive natural compounds in combination with DOX may reshape inflammation by inhibiting changes in gene expression. Collectively, our findings suggest that TA exerts a dual action antioxidant and anti-inflammatory against DOX-induced lung toxicity. It preserves the activity of PPP and antioxidant enzymes, restores GSH levels, and reduces lipid peroxidation and cytokine production. These protective mechanisms may ultimately prevent cellular injury and preserve lung tissue integrity during chemotherapy. The antioxidant effect of TA is multifactorial, involving not only direct ROS scavenging but also the activation and regulation of several key signaling pathways (e.g., Nrf2, MAPK, and NF-κB) and metabolic support for NADPH and GSH homeostasis. These mechanisms provide a comprehensive understanding of how TA mitigates DOX-induced oxidative and inflammatory damage, and they lay a strong conceptual foundation for future translational studies exploring TA as a supportive agent in chemotherapy.

The addition of machine learning–based feature attribution to the biochemical dataset gave mechanistic information to the traditional statistical comparisons. The SHAP analysis of Fig. 5A has indicated clearly that the exposure to DOX causes a typical metabolic and molecular signature whereby it causes the changes in pentose phosphate pathway activity, the inhibition of the antioxidant defenses, and the over-activation of the inflammatory cytokines. SHAP contributions of 6PGD activity, G6PD and 6PGD mRNA, GPX and CAT activity, and MDA were high which means that oxidative balance is severely disrupted after DOX treatment. The dominance of the IL-6 and TNF-alpha mRNA also indicates a redox-regulating loop of amplification in association with inflammation. Combined, these findings indicate that the DOX toxicity is not determined by one pathway but is caused by a complex breakdown of the PPP-dependent production of NADPH, damage to ROS detoxification potential, and pro-inflammatory signaling.

This hierarchy of biomarkers was independently confirmed by permutation importance analysis (Fig. 5B), which provided very high overlap with SHAP-derived importance scores. The similarity of IL-6, GPX, 6PGD, CAT, GSH, and MDA in the list of the highest-ranking variables in the two frameworks makes the use of these variables critical to mechanistic predictors of DOX-related injury. This overlap between two orthogonal feature-importance approaches significantly improves the strength of the results, and it diminishes the chances that the detected rankings of importance are the results of model structure or sampling bias. Notably, the putative use of the enzymes of PPP (G6PD and 6PGD) in the two studies was found as a central indicator that the oxidative stress caused by DOX overwhelms the compensatory mechanisms of NADPH regeneration-driving pathways, which further aggravates the process of lipid peroxidation and the release of cytokines.

The high predictive power of the machine learning–based classifier a small OOB Brier score further confirms that the discovered biomarker profile is a true indication of actual physiological perturbations and not due to a model overfit. The ability of the model to make computational predictions that are consistent with established biochemical mechanisms of DOX toxicity supports the biological validity of the model. In addition to this, the SHAP explanations showed effects of individual features at the individual-sample level showing that the co-treatment of DOX + TA continually suppressed the pathogenic SHAP signatures of PPP disruption, oxidative stress, and inflammation. The PCA measures show that DOX causes the global change in biochemical homeostasis, and the multivariate profile of the results is clearly separated between the control group and the DOX results. This transition occurs as a result of coordinated alterations in antioxidant defense, cytokine and oxidative damage. The observation that TA and DOX + TA clusters are closer to controls indicates TA infers a system-wide protective action as opposed to working on isolated pathways. The heatmap analysis shows mechanistic interactions between oxidative damage and inflammatory signaling. DOX enhanced the relationships between MDA and IL-6/TNF-2, as earlier reports have found that lipid peroxidation enhances the expression of cytokines through the NF-kB pathways. Recovery of the normal patterns of correlation in the TA-treated groups suggests that the compound breaks this pathological feedback process.

The network topology analysis was able to give new understanding of biochemical disorganization caused by DOX. The degradation of the connection between antioxidant nodes indicates the loss of enzymatic control, which is one of the characteristics of redox imbalance. The tightened inflammatory margins in the DOX cluster on the other hand are a manifestation of the development of an undesirable pro-oxidant network. TA helped restore these topological anomalies, enhancing the antioxidative module and reducing the edges of inflammation. This reorganization of the system is supportive of the hypothesis that TA has pleiotropic effects on redox metabolism as well as inflammatory signaling. This confirms the cardiopulmonary or hepatic oxidative injury which is caused by DOX and is prevented by TA. Taken together, these results demonstrate that the joint application of interpretable machine learning instruments and enzymatic/molecular endpoints can be viewed as an effective set of tools to understand the mechanism of DOX toxicity and to assess the potential of antioxidant interventions to be used as a therapy.

## Limitation

This study has several limitations that should be considered when interpreting the findings. First, histopathological evaluation of lung tissue was not performed, as the available samples were allocated to biochemical assays and RNA-based analyses; therefore, morphological confirmation of tissue injury could not be provided. Second, the study tested a single dose of tannic acid (50 mg/kg, i.p.), selected based on prior literature, and thus a formal dose–response relationship and therapeutic window could not be established. Third, only male rats were included to reduce hormonal variability; consequently, potential sex-dependent differences in DOX toxicity and TA-mediated protection cannot be excluded. Fourth, while animals were monitored daily and no mortality was observed, systematic longitudinal body-weight trajectories and standardized clinical scoring were not designed as primary endpoints in this workflow. Finally, the machine learning analyses were used as complementary, integrative tools to prioritize key predictors; however, the dataset size and single-cohort design warrant independent replication for broader generalizability.

## Conclusion

In summary, DOX administration induced a clear oxidative and inflammatory response in rat lung tissue, as reflected by altered redox status, antioxidant enzyme activity, and increased pro-inflammatory cytokine expression. Importantly, TA (50 mg/kg, i.p.) co-treatment attenuated these DOX-induced changes, supporting its protective potential against DOX-related pulmonary toxicity. The complementary machine learning analysis further highlighted a compact set of markers that best distinguished the experimental groups, providing an integrative view of the injury and protection signatures. Future studies should incorporate histopathology, dose–response assessment, and both-sex cohorts to strengthen translational inference.

## Data Availability

All source data for this work (or generated in this study) are available upon reasonable request.
